# Part A: Biodegradable Bio-Composite Film Reinforced with Cellulose Nanocrystals from *Chaetomorpha linum* into Thermoplastic Starch Matrices

**DOI:** 10.3390/polym15061542

**Published:** 2023-03-20

**Authors:** Taghreed Alsufyani, Nour Houda M’sakni

**Affiliations:** 1 Department of Chemistry, College of Science, Taif University, P.O. Box 11099, Taif 21944, Saudi Arabia; 2 Laboratory of Interfaces and Advanced Materials (LIMA), Faculty of Science, Monastir University, Monastir 5019, Tunisia

**Keywords:** green macroalga, *Chaetomorpha linum*, Red Sea, cellulose nanocrystals, thermoplastic starch, bio-composite films, biodegradable

## Abstract

In recent years, macroalgae and microalgae have played a significant role in the production of organic matter, fiber, and minerals on Earth. They contribute to both technical and medicinal applications as well as being a healthy and nutritious food for humans and animals. The theme of this work concerns the development and exploitation of *Chaetomorpha linum (C. *linum)** biomass, through the elaboration of a new starch-based composite film reinforced by cellulose nanocrystals (CL-CNC) derived from C. linum. The first step involves the chemical extraction of CL-CNC from dry *C. linum* algae biomass. To achieve this, three types of cyclic treatment were adopted: alkalinization (sodium hydroxide) followed by bleaching (sodium hypochlorite) and acid hydrolysis (hydrochloric acid). We then studied the optimization of the development of bio-composite films based on corn starch (CS) reinforced by CL-CNC. These polymeric films were produced using the solution-casting technique followed by the thermal evaporation process. Structure and interactions were modified by using different amounts of glycerol plasticizers (20% and 50%) and different CS:CNC ratios (7:3 and 8:2). These materials were characterized by UV visible (UV/Vis), Fourier Transform Infrared (FTIR) and Scanning Electron Microscope (SEM) spectroscopy to understand structure-property relationships. The result revealed that the best matrix composition is 7:3 (CS: CL-CNC) with 50% glycerol, which reflects that the reinforcing effect of *CL-CNC* was greater in bio-composites prepared with a 50% plasticizer, revealing the formation of hydrogen bonds between CL-CNC and CS.

## 1. Introduction

Today, sustainability and environmental issues remain priorities for research firms [1,2], which explains the recent growing interest in research in new areas of application based on non-oil structural materials such as starch [3] and cellulosic materials (crystalline cellulose and amorphous cellulose), which are naturally renewable, biodegradable, and environmentally friendly polymers [2,4]. Starch is a biopolymer, which has the advantage of being plasticized by thermos-mechanical treatment in the presence of water and a plasticizer, such as glycerol, to produce thermoplastic starch. This biodegradable matrix is very hydrophilic, with mediocre mechanical properties [5]. To overcome these drawbacks, solid strengthening charges are mixed with starch during processing. These reinforcement fillers include clay, talc, silica, fiberglass, carbon black, and natural fibers derived from biomass. However, natural fibers have shown a remarkable impact on bio-composite technology because of the nature of cellulose and its crystallinity. Thus, cellulose nanocrystals (CNC) are considerably stronger and more rigid than cellulose nanofibrils (CNF) and native cellulose itself, and are regarded as a better-reinforcing agents than native cellulose [5]. However, starch-based bio-composites have poor mechanical, thermal, and barrier properties relative to polyester films. In addition, these properties are highly permeable to moisture and are reinforced over time due to degradation processes. For these reasons, starch-based bio-composites are more focused on short-term applications. Consequently, it is essential to make some improvements to these properties in order to obtain materials suitable for each application [5]. 

The amylose/amylopectin ratio has a strong quality relationship to the thermal, mechanical, and barrier properties of biofilms [6]. Several studies have shown that the lower the level of amylose (20% for the potato) in the bio-film, the better its mechanical properties and water solubility, and the lower its oxygen and water vapor permeability, while the optical properties of bio-films have an impact in terms of the amylose/amylopectin ratio. In fact, the potato (lower amylose) film was transparent, whereas the corn (27%) and wheat (25%) films were opalescent [6,7]. Therefore, the use of starch has been restricted due to its low performance as well as its fragility, high sensitivity to water and retrogradation, gas and humidity permeability, high viscosity, and limited solubility [8]. Furthermore, plasticizers, chemical modifications, and nanofillers, such as starch nanoparticles, metallic nanoparticles, nanoclays, nanofibers, and others, have been used to improve the properties of starch. CNCs are one of the widely-used nanoscale fillers among starch polymer nanocomposites that strengthen and improve mechanical strength and flexibility with their large surface area, good mechanical properties, and low cost [9,10]. They have also been used to reinforce fragile conductive polymers for use in sensors, batteries, conductive additives, energy storage [11], etc. 

The extraction of microcrystalline cellulose (MCC) from cellulose fibers can be achieved using three types of processes: the mechanical treatments (e.g., homogenization, grinding, and milling), the chemical treatments (e.g., TEMPO oxidation), and the combination of chemical and mechanical treatments [12]. Several studies were carried out to extract MCC from various biomass sources using a chemical process by cyclic alkali treatments and bleaching [13]. The depolymerization process was applied for the extraction of MCCs from sugarcane bagasse using both hydrogen peroxide (H_2_O_2_) and sulfuric acid (H_2_SO_4_) after a series of grinding, autoclaving, alkaline (NaOH), and bleaching (NaClO_2_/CH_3_COOH) treatments. However, CNC and CNF are by-products of MCC resulting from the isolation and extraction of biomass using chemical, physical, and enzymatic processes [14]. On the other hand, the mechanical treatment preserves the non-crystalline MCC parts of the microfibril as well as the length of the fibrils and promotes the CNF isolation, but not for the CNC [12,15]. CNC has also been successfully isolated from the resultant MCC by acid hydrolysis, and it showed an increase in crystallinity due to the removal of amorphous regions present in MCC [16]. CNCs have usually been prepared by acid hydrolysis of wood or cotton pulp, agricultural coconut waste [17], bananas [18], or sugar cane [19]. Typically, CNCs are extracted using sulfuric acid [20] or hydrogen chloride solutions [21] in combination with a subsequent ultrasound or homogenization step at elevated temperatures. 

Sulfuric acid (H_2_SO_4_) is the most common acid for the preparation of nanocellulose by chemical hydrolysis, but other acids can be used, too. Acid hydrolysis of the biomass is generally implemented to obtain stable CNC aqueous suspension [16]. Several chemical hydrolysis acids are used for the modification of the surface polymer chain of CNC with different functional groups to give different surface characteristics. However, the surface modification of CNC depends on the isolation process and further treatment of the nanocrystals. As examples of different functional groups attached to the surface cellulose polymers, sulfite ion shows the surface polymer chain after H_2_SO_4_ hydrolysis, hydroxyl after hydrochloric or hydrobromic acid (HCl or HBr) hydrolysis, dihydrogen phosphate ion after phosphoric acid (H_3_PO_4_) hydrolysis, ammonium ion and hydroxyl group after H_2_SO_4_ hydrolysis followed by a surface cationization, and carboxylate ion after an HCl/HBr hydrolysis followed by a TEMPO-oxidation [15]. However, Ilyas and Sapuan [22] mentioned that CNC, which acted as a reinforcing filler for starch-based polymers, was ideal due to the presence of abundant surface hydroxyl groups on the CNC surface, which are responsible for hydrogen bonding between the non-polar matrices and –OH groups of hydrophilic polymer matrices. 

Polymer matrices used to prepare composites reinforced with CNCs could be divided into two parts––biodegradable and non-biodegradable polymers [23]. Hence, natural polymers including derivatives of cellulose, starch, natural rubber, and biopolymer derivatives, such as Poly-hydroxy-alkanoates (PHA), Polylactic acid (PLA), Polycaprolactone (PCL), etc.), were commonly used as biodegradable polymers for preparing bio-nanocomposites reinforced with nanocrystals of cellulose [23,24]. Apart from favorable physico-chemical and mechanical properties, the most important requirement for a biodegradable polymer to be used for medical applications is its biocompatibility and the non-cytotoxicity of its degradation products. For this reason, the objective of this work was to produce starch films reinforced with CNC and determine their degree of biodegradation with the enzymatic method in order to propose alternative materials to reduce the excessive use of petroleum derivatives.

## 2. Experimental Work

### 2.1. Materials

Macro-algae thalli belonging to the order Cladophorales, *Chaetomorpha linum* were used as raw material to prepare cellulose nanocrystals. Green algae were harvested on the southwest coast of the Red Sea in Jeddah KSA (coordinates 21°37′41″ N and 39°6′11″ E). The vegetable source (corn) used to make the starch was purchased from the merchant in Taif (Saudi Arabia). Starch-based plasticizers were typically used at a weight rate of 30%. Hydrochloric acid (HCl), sodium hydroxide (NaOH), sodium hypochlorite (NaClO), and glycerol (C_3_H_8_O_3_) were obtained from VWR, PROLABO (Lutterworth, UK). All chemicals used were of analytical grade. Mixture enzyme was formed by α-amylase from human saliva (type XIII-A, lyophilized powder, 300–1500 units/mg protein) and glucoamylase from *Rhizopus sp* (lyophilized powder, 30–60 units/mg protein (biuret), ≤0.02% glucose) was obtained from Sigma Aldrich (St. Louis, MO, USA).

#### 2.1.1. Extract of Chemical Cellulose Nanocrystals 

**Figure 1:**
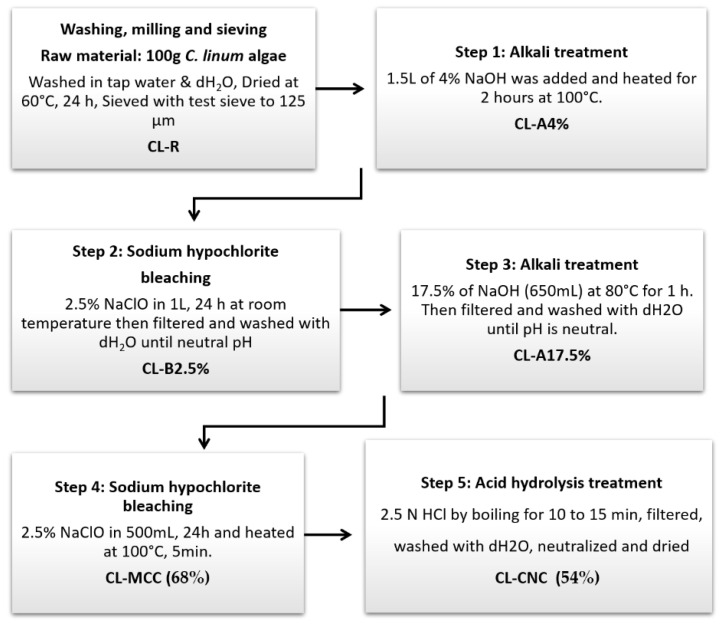
Schematic diagram of cellulose nanocrystals separation from *C. linum*.

Nanocrystalline cellulose was isolated from the marine biomass *C. linum* following the method discussed by Lubis, et al. [25], with slight modification. Three different types of treatment have been adopted with *C. linum*, namely, alkalinization (NaOH), bleaching (sodium hypochlorite), and acid hydrolysis treatment (HCl), as shown in Figure 1. The obtained thalli were rinsed once with a tap and twice with distilled water to avoid any impurities, dried at room temperature, and then at 60 °C for 24 h until the mass was stabilized. Lastly, dried thalli was ground into fine powder, served (granulometry less than 125 µm), and stored in glass containers (CL-R).

100 g of algae powder (CL-R) was subjected to alkalinization by inserting the fine powder into a glass beaker, and 1.5 L of 4% NaOH was added and then heated for 2 h at 100 °C. The residues were filtered out and washed with deionized water at a neutral pH (CL-A 4%). This step was followed by the bleaching treatment with 2.5% NaClO soaked in 1L for 24 h at room temperature, and then filtered, with the residue washed with deionized water to neutral pH (CL-B 2.5%). After bleaching, 17.5% of NaOH (650 mL) was added to the residue and heated at 80 °C for 1 h, filtered, and washed (CL-A 17.5%). Finally, residues were bleached using 2.5% NaClO (500 mL) and heated for 5 min at 100 °C. The resulting residue was filtered, washed (up to pH = 7), and oven dried at 40 °C for 96 h to obtain microcrystalline cellulose CL-MCC residue.

The microcrystalline cellulose powder (CL-MCC) obtained was hydrolyzed using 2.5 N HCl by boiling for 10 to 15 min and filtered. The obtained residue is neutrally washed in distilled water, dried, dialyzed, and mushed to a fine powder labeled CNCs (CL-CNC). The yield (%) of CL-MCC and CL-CNC obtained was calculated according to the following Equation (1) [26]:

(1) $$\mathrm{Yield}\;\%=\frac{m_{f\;}\cdot \;V_{1}}{m_{i}\;\cdot \;V_{2}}\times 100\;$$

where $m_{f\;}$ (g) is the mass of the vacuum-dried sample, $m_{i}$ (g) is the initial mass of the dry cellulose sources, $V_{1}$ (mL) is the total volume of the sample suspension after dialysis, and $V_{2}$ (mL) is the volume of the sample suspension that was vacuum dried. The yield achieved for CL-MCC and CL-CNC was found to be 68% and 54%, respectively.

#### 2.1.2. Starch Extraction

100 g corn was cut to a size of approximately 2 cm and mashed with water in a blender. The blended starch-rich suspension was then filtered through Whatman No. 1 (Whatman, UK) filter paper to get wet starch. The residue was dried in the oven for 30 min at 70 °C. Corn-S (CS) starch yield was calculated and found to be 20%. 

#### 2.1.3. Development of Bioplastic Synthesizers from Extracts of Cellulose Nanocrystals and Starch 

The bioplastic films were prepared from the reinforced thermoplastic starch using CNC prepared as described above from the *C. linum* algae biomass, with the use of the variable glycerol mass that played a major role in the appearance, stiffness, and elasticity of the biofilms. In the production of biofilms, the technique of “casting-evaporation” was adopted in which the macromolecules were continuously dissolved in the solution and the solvent was evaporated to produce a solid biofilm [27]. Through this technique, simple and inexpensive biofilms could be produced at the laboratory scale, with improved properties comparable to those obtained by the wet process (such as dipping–molding).

Two films were synthesized by varying the CS:CL-CNC ratio (7:3, and 8:2) by preparing two solutions:

(1) A thermoplastic starch solution by dissolving the starch (CS) in water with a ratio of 1:20 (starch: distilled water) and boiling for 10 min at 80 °C. Totals of 20% and 50% glycerol were then added to the dissolved starch mass. 

(2) A solution of CNC (CL-CNC) was prepared by dissolving a specific weight of cellulose in 40 mL of 5% NaOH and stirring overnight.

The prepared solutions (Table 1) were thus mixed by adding the CL-CNC solution to the thermoplastic starch solution, and homogenized for 20 min with continuous stirring and heating at 80 °C. The prepared solution was cooled and imprinted on a 25 × 25 × 3 mm acrylic mold. The bio-composite was kept in the oven (60 °C) for drying for 24 h and then removed from the mold.

### 2.2. Characterization Methods

#### 2.2.1. UV-Visible Analysis

The UV-Visible analysis was performed on a dual-beam Lambda 35 spectrophotometer (Perkin Elmer, Waltham, MA, USA) with a wavelength (ʎmax) range of 200 to 800 nm using a quartz cuvette with a 1 cm optical path. For optical properties, the UV-Vis-NIR (JASCO; V670, Easton, Portland, OR, USA) spectrophotometer was used to study the optical transmittance (%) of films prepared over the wavelength range of 190 nm to 900 nm. 

#### 2.2.2. FTIR Analysis

Several stages involved in the development of bio-composite films and crude algae were studied by FTIR (Thermo spectrophotometer, Nicolet IR 200, Madison, WI, USA). FTIR spectrums were recorded between 4000 and 400 cm^−1^ and compared with data already reported to distinguish the signal in a specific manner.

The in-depth analysis of the FTIR spectrum combined with the information collected in the literature makes it possible to distinguish the signals in a very specific manner. However, the exceptional FTIR region for the detection of cellulose crystallinity ranged from 850 to 1500 cm^−1^ [28,29]. The order index is defined by O’Connor et al. [29], as the absorbance ratio of the bands detected at 1430 and 900 cm^−1^, known as crystalline and amorphous cellulose bands, occurring as a result of CH_2_ vibration (the symmetric and rolling bending, respectively), and it is also defined as LOI: A1430/A908 [28]. The absorbance ratio of the bands detected at 1326 and 2903 cm^−1^ was attributed to the flexural and stretching vibrations of the C-H bonds. This relationship corresponds to the total crystallinity index (TCI: A1326/A2903) [28]. The hydrogen bonding intensity was reported in previous research in the form of HBI ratio (HBI: A3350/A1326) [28,30]. 

For the water content calculation (Herrera-Gómez et al., 2001), IR spectra were plotted by using Sigmaplot (Systat Software Inc., version 12, Bayshore, San Jose, CA, USA).

The total area under the curve (AT) as well as the area in the region from 1560 to 1800 cm corresponds to the bending vibration mode of the water molecules contained in the film (AW), and were computed by macros (see Supplementary Figures S3 and S4).

The percentage of the water content is equal to (AW/AT) × 100.

#### 2.2.3. SEM Analysis

JEOL model JEM-2000FX (Tokyo, Japan) instrument operated at an accelerating voltage of (15–25) Kilo voltage used to determine SEM (scanning electron microscope), EDX (energy dispersive X-ray spectroscopy), and TEM (transmissions electron microscopy) measurements.

SEM and EDX images were taken for the characterization of the morphology and the microstructure of all materials obtained at different stages of the bio-nanocomposite film development process (see Supplementary Figure S1).

#### 2.2.4. Enzymatic Degradation

The first step of this study was to test the degradation of a known starch solution by (α-amylase and glucoamylase) enzymes. The objective of this test is to determine the conditions (time, concentration, and temperature) favoring the proper functioning of the enzyme in order to degrade a starch solution of a specific concentration. The attached table (see Supplementary Table S1) summarizes the results. The second step was to study the biodegradability test at the favoring conditions of starch-based bio-composite film reinforced with CNC at two rates (7:3 and 8:2) and commercial bag film, which was used as a reference formed by PEHD. 

Step 1: For testing the condition of activity of the mixture enzyme, a primary test was realized for the degradation of a known starch solution (10 g/L) by the mixture of enzymes αamylase (α-amylase and glucoamylase) with activity 31.6 FPU/mL. From a corn starch solution of the concentration C = 10 g/L, eight test tubes were filled with starch, distilled water, and a mixture of enzymes (α-amylase and glucoamylase) (0, 0.2, and 0.5 mL) as described in the table below (see Supplementary Table S1). The tubes were incubated at different temperatures (0, 37, and 50 °C). From this moment, the time, t = 0 min, was noted. Every hour, two samples of each tube have been tested: one will be placed in a cell of the multi-well plate to which a drop of iodized water is added, and the other will be placed in a test tube to which three drops of Fehling liquor are added and heated for 1 min. 

Step 2: Biodegradation of the elaborated and reference films was measured in laboratory conditions by enzymatic hydrolysis. The measurements were carried out using an enzyme mixture that contained two different commercial enzyme preparations (α-amylase and glucoamylase). The activity of the prepared enzyme mixture was 31.6 FPU/mL (filter paper unit/mL). The dried films were cut into dimensions 2 × 2 cm^2^, and then the samples were weighed accurately using a digital balance (masse = 0.75 mg) and the enzymatic mixture was immersed in 50 mL falcon tubes. 0.1 M sodium acetate buffer (pH 8.5) was added to the falcon tubes containing the film samples. The incubation was started with the addition of the enzyme mixture, and the test tubes were placed in a 50 °C water bath with mixing. The test of biodegradability according to the (α-amylase and glucoamylase) enzymes was released for the biodegradable bio-composite already prepared with an 8:2 and 7:3 ratio CS:CL-CNC, and for the petrochemical PEHD film from the industry as a reference (sample with no cellulose). 10 mL of buffer solution and 0.5 mL of mixture enzyme were added at zero time. Three replicates of each sample were measured. The tubes were incubated at zero time in a T = 50 °C water bath. The hydrolysis time was constant at 48 h. A 2 day incubation time was chosen for this study as it was shown to be enough for the degradation of pure cellulose samples (see Supplementary Table S1). Time-dependent sampling was then carried out for each solution to determine the amount of sugar reduction after 12, 24, and 48 h of contact with the enzyme mixture.

After the hydrolysis time with the enzyme mixture, reducing sugars are determined by a colorimetric method with the 3,5-dinitrosalycilic acid (DNS) reagent as described by Miller [31]. It is a non-stoichiometric oxidation-reduction reaction, allowing the quantification of reducing sugars. In an alkaline and hot medium, the DNS, initially yellow in color, is reduced by reducing monosaccharides to orange–red (3-amino-5-nitrosalycilic acid). The absorbance of oxidized DNS is read at λ = 546 nm. The calibration curve (see Supplementary Figure S5) was obtained by plotting the absorbance values of the standard solutions at λ = 546 nm as a function of their α-D-glucose concentration. The degree of hydrolysis of the cellulose samples was calculated by Equation (2) by comparing the reducing sugar content to the initial amount of cellulose.

(2) $$\mathrm{Degree}\;\mathrm{of}\;\mathrm{hydrolysis}\;\left(\%\right)=\frac{\mathrm{reducing}\;\mathrm{glucose}\;\mathrm{content}\;\left(\mathrm{mg}/\mathrm{mL}\right)}{\mathrm{initial}\;\mathrm{amount}\;\mathrm{of}\;\mathrm{cellulose}\;\left(\mathrm{mg}/\mathrm{mL}\right)}$$

## 3. Results and Discussion

### 3.1. Characterization of Cellulose Nanocrystals Extract

**Figure 2:**
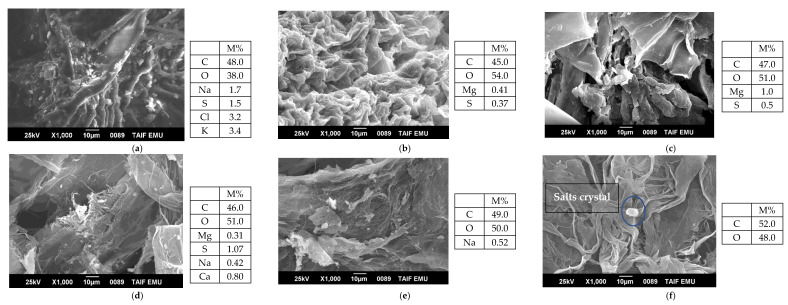
SEM and EDX analysis (see Supplementary Figure S1) showing various steps of cellulose nanocrystals production from *C. linum* with 1000 × magnification and operated at an accelerating voltage of 25 KeV. (**a**) w material: *C. linum* (CL-R) (Fine powder); (**b**) lkalinization treatment (CL-A4%) (4% NaOH); (**c**) Bleaching treatment (CL-B2.5%) (2.5% NaClO); (**d**) Alkalinization treatment (CL-A17.5%) (17.5% NaOH); (**e**) Bleaching treatment (CL-MCC) (2.5% NaClO); (**f**) Acid hydrolysis treatment (CL-CNC) (2.5N HCl).

The microcrystalline cellulose (CL-MCC) was extracted and purified by alkaline treatment followed by bleaching using, respectively, NaOH and NaClO to give a yield of 54%. The treatment of pure acid hydrolysis of the cellulose microfibril by HCl filled the amorphous regions of microfibrils under certain conditions to obtain the CNCs (CL-CNC), which were white [32], and which were also characterized by SEM, EDX, and FTIR, as shown in Figure 2a–f and Supplementary Figure S2a–d, respectively.

Cellulose exhibits two different phases, amorphous and crystalline, in which the concentration distribution of the crystalline phase is more perpendicular than the amorphous phase. To construct the fibrous structure, cellulose was incorporated into a hemicellulose-lignin matrix of several compositions that differ from one another, called natural composites [33]. However, an alkaline treatment with NaOH was the most widely-used chemical treatment of CNCs in biomass, while the removal of lignin, hemicellulose, and impurities from plant biomass took place. The interfibrillar region becomes less dense and rigid, which allows fibrils to rearrange. The treatment increases the extraction of the fiber as well as increasing the contact surface between the cellulose and the matrix. If the alkaline treatment was not appropriately optimized, the fibers could deteriorate or be damaged. 

Therefore, time, temperature, and concentration influence the optical, mechanical, and thermal properties of the fibers [34]. Alkaline treatment also influences the thermal and physical properties of natural fibers as well as the morphology and size of the fibers [35]. However, bleaching was carried out as a preparatory step for coloring and producing uniform white CL-CNC, and several reagents, such as hydrogen peroxide, sodium chlorite, and sodium hypochlorite, could be used for their prescribed purposes.

With the acid hydrolysis, the cellulose microfibril filled the amorphous regions of the microfibrils to obtain the cellulose nanocrystals. Hence, these microfibrils were broken down into shorter crystalline parts with a high degree of crystallinity, generally referred to as CL-CNC, as shown in Figure 2f and Figure S2 [36].

#### 3.1.1. Morphological Analysis of Cellulose Nanocrystals and By-Products during Extraction

The chemical composition of the biomass of *C. linum* (CL-R) and the various derivatives obtained during the protocol for the separation of CL-CNC from *C. linum* were evaluated by SEM and EDX analysis. The separation of the CL-CNC confirms whether the homogenization process was successful in converting cellulose into individual fibrils and then into nanocrystals, or not. Morphological examination by SEM analysis showed that the texture of (CL-R) was a rough surface containing cellulose in the form of coiled and soft fat with rough cores (Figure 2a and Figure S1). With a 4% alkalization treatment (CL-A 4%), a smooth surface with white extract was deposited (Figure 2b and Figure S1). Using an alkaline solution causes the intra-crystalline swelling of the fibers, making the hydroxyl sites internal to the fibers accessible for chemical modifications [37,38,39,40]. The solvents used are non-derivatizing, i.e., do not chemically modify the cellulose [41].

The alkaline treatment of the biomass of *C. linum* resulted in the partial elimination of hemicellulose and lignin from the fiber’s surface [40]. The surface area of the *linum* fibers was found to be much lighter after alkalinization, which increased the water absorption (Figure 2d and Figure S1). After immersion in the 2.5% NaClO-containing solution, the white extract was removed. A distinct and smooth surface was observed on the biomass, thus forming (CL-B2.5%) (Figure 2c and Figure S1). 

After the second alkalinization (17.5% NaOH), the derivative CL-A17.5% showed a rougher, sharper, and more crystalline and amorphous appearance of the fibers. These fibers remained sharper and more crystalline after the second bleaching treatment, using 2.5% NaClO to give microcrystalline cellulose (CL-MCC), as shown in Figure 2e and Figure S1. However, Figure 2b–e confirmed the self-assembled structures of cellulose fibers, which are due to the strong interfibrillar attraction between the surface and OH groups.

Moreover, the functional properties of cellulose and CNC were linked to the morphological characteristics that depend on the nature of the biomass and the hydrolysis method [42]. The crimped shape gives a large specific surface area, which facilitates the formulation of different composites and hydrolysis [43]. The nanocellulose was produced by a powerful bleaching agent (2.5% NaClO) combined with hydrolysis using 2.5N HCl (Figure 2f and Figure S1), which extracts amorphous cellulose by favoring the fragmentation of cellulose fibers and the hydrolysis of internal fiber regions. 

Figure 2f and Figure S1 confirmed the shape of the CL-CNC determining the flexible, non-fibrous, agglomerated structure [44]. These results can be explained by the fact that the CNC obtained by hydrogen chloride hydrolysis shows the absence of a negative surface charge in the form of sulfate present in the surface charge of sulfuric acid hydrolysis [45]. On the other hand, CNC made from sulfuric acid hydrolysis is more stable than CNC made from hydrogen chloride acid. This can be attributed to the acidic sulfate half-esters groups that are introduced to the surface of the CNC, which generate the double-layer electrical repulsion between the nanoparticles in the suspension. This phenomenon reduces the agglomeration and flocculation of the CNCs in the aqueous medium and also justifies their interaction with themselves and with the polymer matrix [46].

#### 3.1.2. Functional Group of Cellulose Nanocrystals and Its By-Products during Extraction

The structural changes in the different samples obtained during the CNC extraction protocol from the *C. linum* biomass were analyzed by FTIR at various processing stages. The FTIR spectra of the biomass before and after alkaline treatment and bleaching are shown in Supplementary Figure S2a–d.

For the CL-R spectrum (see Supplementary Figure S2a), the absorbance peaks in the regions of 3400–3300 cm^−1^ were recognized as stretching and bending vibrations of the OH groups present in the cellulose, while the band around 2900–2800 cm^−1^ represents the CH stretch [47], similar peak shapes, specifically, for cellulose, lignin, and hemicelluloses [48,49]. A significant peak at 1640 cm^−1^ represents the -C=O stretching vibrations of the amide groups [50]. Moreover, the peaks raised at 1420 to 1430 cm^−1^ were attributed to the bending of CH_2_ and were also related to cellulose I [48], and the band at 1330–1380 cm^−1^ corresponded to the C-H and C-O (bending vibration) of polysaccharides [51]. Among the main absorption bands of interest, the peaks at 1200 and 950 cm^−1^ characterized the absorption of C-O-C vibrations from polysaccharides. However, a small peak at 1161 cm^−1^ was attributed to the asymmetric C-O-C stretching vibrations associated with cellulose I and cellulose II [52]. In addition, a shoulder at 1746 cm^−1^ in the CL-R spectrum was attributed to the acetyl and uronic ester groups of hemicelluloses or the ester bond of the carboxylic group of ferulic and p-coumaric acids of lignin and/or hemicellulose. An absence of signal in the CL-A 4% spectrum indicated that the pre-treatment sodium hydroxide almost cleaved the ester bond from the hemicelluloses and/or lignin [53].

The cellulose-related bands were more intense in the spectra of the samples after treatment, as the removal of the lignin increased their crystalline content. Indeed, the peaks attributed to cellulosic C-H and C-O-C correspondingly extend at 1320 cm^−1^ and 1161 cm^−1^ and were more intense in the CL-A and CL-B samples than in raw biomass (CL-R).

Supplementary Figure S2a,b confirm that the steps of the alkaline treatments have removed most of the lignin from the cellulosic fibers. However, the bands at 1440 cm^−1^ disappeared after the treatments [54], which was absent in CL-A. The peak attributed to the COOH groups appeared in the CL-R spectrum at 1640 cm^−1^ and disappeared during treatment with the NaOH and the sodium polychloride that were applied to the biomass.

By comparing the spectrum before and after the chemical treatment of the biomass used, it was observed that the intensity of the typical peak of C-H groups present in the cellulose at 2889 cm^−1^ is considerably increased [55]. The obtained results confirmed that the effect of chemical treatment using a low concentration of NaOH on the degradation of coating substances (lignin, hemicellulose, and pectin) could influence the size of cellulosic fibers relative to the initial length [56].

Furthermore, a small peak was observed around 1534 cm^−1^ in the CL-R biomass, corresponding to a N-H curvature in the proteins. This peak was not present in CL-CNC, indicating that cellulose nanocrystal samples were protein-free (Table 2). The CL-CNC IR spectrum showed no evidence of strong peaks around 1220 cm^−1^ and 865 cm^−1^. These peaks correspond to the S=O stretch and the C-O-S stretch characteristic of the sulfated polysaccharide specific to algal biomass [57]. The absence of such peaks, therefore, indicates that sulfated polysaccharides were successfully removed from the cellulose fraction by using the chemical treatment protocol [58].

The results confirmed that a chemical treatment using a low concentration of NaOH (4% and 17.5% *v*/*v*) and NaClO (2.5%) was effective in removing the amorphous components of the *C. linum* biomass [56,59]. FTIR analysis indicated that the CL-CNC was effectively extracted with the chemical treatment without any further degradation or formation of side products. The absence of the vibration band located around 1775 cm^−1^ is attributed to the elongated vibrations of the specific C=O bond of the hemicellulose residues on cellulosic chains, or else is due to the likely oxidation in the bleaching stage [60].

**Table 2:** Assignment of bands found in FTIR spectra of isolated CL-CNC, bio-composites films, reported pure glycerol, cellulose, and corn starch.

	Main Peak (cm^−1^) from Isolated CL-CNC and Bio-Composite Films						Main Peak (cm^−1^) from Pure Glycerol		Main Peak (cm^−1^) from Cellulose		Main Peak (cm^−1^) from Corn Starch	
	CL-CNC		CS:CL-CNC7:3-50%		CS:CL-CNC 8:2-20%		Reported Glycerol [61]		Reported Cellulose [44]		Reported Corn Starch (Corn-S) [60]	
	Peak (cm^−1^)	% T	Peak (cm^−1^)	% T	Peak (cm^−1^)	% T	Functional Groups of Glycerol	Λ cm^−1^ of FT-IR Spectrum	Functional Groups of Cellulose	Λ cm^−1^ of FT-IR Spectrum	Functional Groups of Corn-S	Λ cm^−1^ of FT-IR Spectrum
Elongational vibration bands of the O-H link of the primary and secondary alcohol functions	3363	67.60	3446	64.67	3567 3344	89.22 56.72			O–H groups stretching vibration	3347–3450	Stretching of hydrogen-bonded hydroxyl groups.	3410
	-	-	3259	68.40	3169	52.14	Affiliated to O–H bond	3320	-	-	-	-
Elongational vibrations of the C-H bond	2900	89.67	2928	75.91	-	-	C–H stretching vibration	2908	C–H stretching vibration	2897–2990	Axial deformation of the CH_2_ group	2932
Water adsorbed on cellulose	1643	96.28	1677	65.01	1637	69.54	-	-	H_2_O absorbed	1632–1645	Tightly bound water present in hygroscopic materials	1651
Crystallinity of cellulosic materials: Vibration of symmetric bending of CH_2_	1430	88.52	1439	40.76	-	-	-	-	CH_2_ bending vibration	1425–1468	Bending of methyl group	1452
Strain vibrations in the plane of the O-H functions of alcohols	1326	80.91	-	-	1365	31.63	-	-	-	-	C-OH bending vibration of starch molecule	1338
Antisymmetric expansion vibration of the C-O-C glycosidic bond	1161	72.60	1159	47.78	-	-	Stretching vibrations of C–O linkages	1150	C–O–C glycosidic band stretching vibration	1162–1172	Stretching vibrations of C-O bonds in C-O-H of starch molecule	1157
Vibrations of the C–O bond of carbons 2, 3 and 6	1110	58.25	-	-	-	-	Stretching vibrations of C–O in C2	1117	-	-	-	-
	1037	38.23	-	-	-	-	Stretching vibrations of the C–O linkage in C1 and C3	1045	-	-	-	-
Crystallinity of cellulosic materials: Vibration of symmetric bending of CH_2_	1016	47.56	1004	34.53	-	-	Vibration of the skeleton C–C	995	-	-	-	-
	-	-	918	40.82	-	-		925	C–H rock vibration	896–905	C-O-C groups in the anhydroglucose ring within the starch structure	929
Attributed to the amorphous region	908	85.94	-	-	-	-		850				
Associated with the cellulosic β-glycosidic linkages	-	-	841	29.48	843	54.57	Vibrations of C–C linkages	800				860
	-	-	710	54.50	731	48.42	-	-	-	-		763
Attributed to δCOH out of plane [26]	667 612	61.58 53.69	-	-	-	-	-	-	-	-	-	-
Attributed to C6-OH torsion [26]	608	53.70	559	14.46	586	19.18	-	-	-	-	-	-

A thorough, total crystallinity index can be determined by FTIR analysis. The results were shown in both (see Supplementary Figure S2d) and Table 3.

From the supramolecular structure of cellulose, the most exciting parts of the spectrum are between 3700 and 3000 cm^−1^ (specific for the formation of hydrogen bonds) from 1420–1430 cm^−1^ (associated with the rate of the crystal structure of cellulose) and in the region of 900 to 890 cm^−1^ (attributed to the amorphous region) [62]. However, both LOI and TCI parameters are very useful in describing the properties of cellulose (Table 3).

Considering the chain mobility and bond distance, the hydrogen bond strength (HBI) of cellulose is closely related to crystallinity and the degree of intermolecular regularity (crystallinity) as well as the amount of cellulose water content [63]. The TCI is proportional to the rate of crystallinity of the cellulose [64] while the LOI value is correlated to the overall degree of order in the cellulose [64,65]. Cellulose (CL-CNC) revealed the highest TCI, LOI and HBI values (Table 3), indicating a higher degree of crystallinity and a more ordered cellulose structure than those calculated for CL-MCC.

Similarly, vegetable fibers extracted from several vegetable sources have shown that the TCI values for the 10 species vary from 0.237 for the cellulose of *Mezilaurus itauba* to 1.240 for the cellulose of Curaua [65]. 

Hence, in comparison to the LOI values, *Eucalyptus grandis* also showed the highest crystallinity cellulose rate (LOI = 3.172), whereas buriti showed the lowest value (LOI = 0.780). Buriti presented a value (HBI = 2.241) almost twice as high as the other vegetable fibers (HBI = 1.119), having the lowest value. The results indicated that the Hydrolysis Acid (2.5N HCl) of the CL-MCC material favored the increase of the cellulosic crystallinity of the material (CL-CNC) [66] and, subsequently, the modification of the quantity of bound water in the fibrous structure [65]. Therefore, the trend detected in the changes in HBI from 1.014 ± 0.002 to 1.035 ± 0.002 is the origin of cellulose fiber-water molecule interactions. The visible increase could also be explained by the increasing amount of cellulose-water hydrogen bonds or likely due to cellulose-cellulose interactions. This increase was also noted for the value of TCI, which explained a higher and more stable order due to hydrogen bonding [62].

### 3.2. Characterization of a Bio-Composite Film

#### 3.2.1. Functional Group of Bio-Composite Film

Visualization interactions between CS chains, glycerol molecules, and isolated CL-CNC were identified by FTIR spectroscopy. The results of the FTIR spectral analysis of the CL-CNC extract as well as the bio-composite films obtained using the different formulations (CS:CL-CNC 8:2-20%, and CS:CL-CNC 7:3-50%) are shown in Supplementary Figure S3 and Table 2.

The characteristic peaks of starch have determined the intermolecular hydrogen bonds at 3410 cm^−1^, 2932 cm^−1^, 1651 cm^−1^, and 1452 cm^−1^ [60]. Ewelina Basiak et al. illustrated pure glycerol attributing five typical absorption bands located between 800 and 1150 cm^−1^ (Table 2) [61]. Based on the FTIR spectrum of CL-CNC isolated from *C. linum*, the characteristic peaks of cellulose were found at 3363 cm^−1^, 2900 cm^−1^, 1643 cm^−1^, 1430 cm^−1^, 1161 cm^−1^, 1110 cm^−1^, 1036 cm^−1^, 1016 cm^−1^, 908 cm^−1^, 667 cm^−1^, 612 cm^−1^, and 608 cm^−1^, respectively.

In comparison, the corn starch reported by Maréchal and Chanzy [60] and the spectrum of CL-CNC, the profile of the film spectrums of CS:CL-CNC 7:3-50%, and CS:CL-CNC 8:2-20% all undergo a change or disappearance in the bands appearing or in their positions. However, the two peaks of 2900 cm^−1^ (elongation of vibrations of C-H) and 1430 cm^−1^ (symmetric vibration of bond CH_2_, which characterizes the crystallinity of cellulose) broadened with a higher intensity in the film CS:CL-CNC 7:3-50% and disappeared for the CS:CL-CNC 8:2-20% film. The results indicated that starch has a stronger intra-molecular interaction with CL-CNC in the CS:CL-CNC 7:3-50% film than in the CS:CL-CNC 8:2-20% film. Compared with the results of Ewelina Basiak et al., the most important inter-molecular interaction affecting the properties of bio-composites is hydrogen bonding, whereas the change in the composition of the matrix favors the change in the network of hydrogen bonds [61].

The broadband present in the range 3410–3300 cm^−1^ is attributed to the stretching vibrational mode of the O–H bond, specific to inter- and intra-molecular hydrogen bonds in starch and starch (cellulose) structure. The intensity of the band is higher in the film CS:CL-CNC 7:3-50% (64.67%) than that of CS:CL-CNC 8:2-20% (56.35%), and this increase is observed due to the plasticization of starch chains linked to the formation of hydrogen interactions between starch molecules and glycerol [3]. The same phenomenon was detected in several studies by the incorporation of CNCs into the matrix containing starch [67,68]. However, the higher the amount of CL-CNC, the more intense the absorption band obtained in the biofilms.

The band at 1651 cm^−1^ demonstrates the bending vibrational mode of the O–H bond in the water molecules, which is adsorbed in the amorphous regions of starch [3]. A similar band was observed for the CL-CNC at 1671 cm^−1^, with a higher intensity than in the biofilms, and the decrease in the intensity of the band was attributed to the variations in the crystallinity of the starch [3]. The incorporation of starch leads to a decrease in the intensity of the band, indicating a lower number of water molecules intercalated in amorphous regions. The infrared spectrum in the region of C–C and C–O bond stretching vibrational modes (1300–800 cm^−1^) became sensitive to structural changes due to starch—CNC interactions as discussed by Jeroen et al. [69].

The effect of glycerol/CS/CL-CNC interactions was analyzed by comparing the absorbance bands of pure glycerol to the spectra of bio-composites with 20% and 50% glycerol. As shown in Supplementary Figure S3 and compared to the IR spectra of the reference samples (pure glycerol, Table 2), the characteristic carbohydrate peak (1161 cm^−1^) was shifted to C–O stretching and moved to 1159 cm^−1^ (film with 50% glycerol). In the 20% glycerol film, the peak disappeared by confirming that with 20% glycerol and an 8:2 (CS:CL-CNC) ratio, the interaction was not effective. Moreover, the characteristic peak at 2908 cm^−1^ of pure glycerol (due to C–H vibrations) was shifted to 2928 cm^−1^ for the films composed of 50% glycerol and almost absent for the film composed of 20% glycerol. The peak at 3320 cm^−1^ (affiliated to the O–H bond) corresponds to the appearance of new peaks, respectively, around 3259 cm^−1^ and 3169 cm^−1^ for both films (50% and 20% glycerol). These changes indicated that the addition of glycerol promoted hydrogen bonding interactions between starch, glycerol, and CL-CNC, which was most apparent in the film composed of 7:3 (CS:CL-CNC) and 50% glycerol. The results concluded that the plasticizing effect of the glycerol promotes the hydrogen interaction between the different biocomponents of the bio-composite matrix, which is due to the hydrophilic nature of glycerol and starch [70].

#### 3.2.2. Morphology of Obtained Bio-Composite

The observation of the surface of bioplastic films based on CL-CNC derived from *C. linum* and starch derived from corn in the presence of glycerol was carried out by SEM to follow the modification of the surface of the samples in the presence of starch and glycerol.

Based on the apparent morphology of the films, CS:CL-CNC 7:3-50% was found transparent and flexible while CS: CL-CNC 8:2-20% was opaque and brittle. According to the FTIR results, the matrix of the film CS:CL-CNC 7:3-50% was improved by preparing a bio-nanocomposite intercalating with variable amounts of green AgNPs from *C. linum* biomass. The optical properties of the film were improved to apply on an industrial scale. 

**Figure 3:**
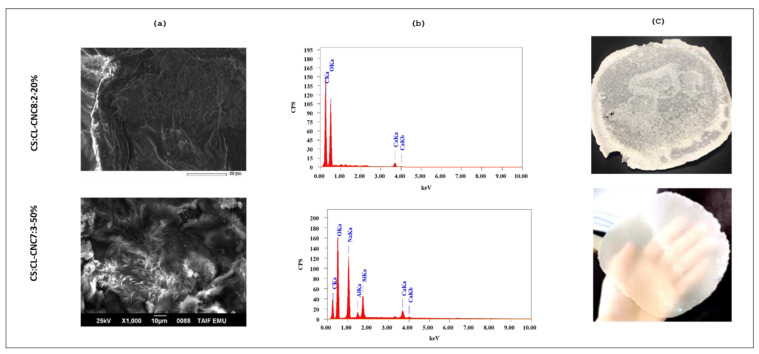
(**a**) SEM, (**b**) EDX, and (**c**) real photo of bio-composites films CS:CL-CNC 8:2-20% (brittle and broken film) and CS:CL-CNC7:3-50% (compact and flexible, see Supplementary Video S1) with magnification 1000×.

Figure 3a,b represents the SEM/EDX images on the surface morphology of (1) CS: Cl-CNC 8:2 bio-composite film containing 25% by weight of CL-CNC, and (2) bio-composite film of CS:Cl-CNC 7:3 containing 42.85% by weight of CL-CNC, both successively containing 20% and 50% by weight of glycerol as a plasticizer. The morphology of the biofilms determined the presence of cone-shaped filaments in the bioplastic CS:CL-CNC 7:3-50% containing several pieces of microfibers, while the bioplastic CS:CL-CNC 8:2-20% configured the smooth and porous presence of spores due to the high starch content (80%) compared to CL-CNC (20%). The difference in morphology was probably due to the incomplete dissolution of the CL-CNC fibers in the presence of the low level of glycerol 20% in the bioplastic CS:CL-CNC 8:2-20% compared to that of CS:CL-CNC 7:3-50%, which led to a homogeneous mixture of microfibers and nanofibers. In such cases, the nanofibers (most likely the starch and/or cellulose particles) filled the pores of neighboring microfibers (most likely cellulose microfibers), making the hybrid film densely compact and more rigid [71,72]. Likewise, the microscopic surface structure of CS:CL-CNC 8:2-20% film indicated the presence of unplasticized starch granules. In general, glycerol was well used to plasticize starches with a high amylose content, such as corn starch [61,73,74]. Nevertheless, some researchers recommended that the plasticizer concentrations >30% by weight completely dissolved the starch granules and thus obtained homogeneous starch films with a smooth surface [61,73,75]. Figure 3c has shown that the texture of CS:CL-CNC 7:3-50% is flexible and transparent, whereas CS:CL-CNC 8:2-20% is brittle and opaque.

#### 3.2.3. Water Content of the Synthesized Bio-Composites Films 

All bio-composite materials can absorb water in humid environments or submerged in water. Natural fibers with a hydrophilic character due to hydroxyl (–OH) and other polar groups in their different constituents such as cellulose and hemicellulose are interested in water absorption [76,77]. This phenomenon leads to swelling of the fiber, degradation of a fiber-matrix interface, a plasticizing effect, and an expansion of the space between the fiber bundles that reduces the efficiency of charge transfer and leads to decreased bio-composite performance and reduced mechanical properties [77]. Therefore, an inevitable step before the use of bio-composites in each application is to analyze the water intake of the developed bio-composite. Water uptake in composites can be affected by fiber volume fraction, matrix viscosity, voids, moisture, and temperature [76,78].

The IR absorption spectrum (see Supplementary Figure S4, Table S2) in the region from 1580 to 1700 cm^−1^ corresponds to the bending vibration mode of the water molecules contained in the film. The peak area (Aw) corresponding to IR water uptake increases as expected as Aw increases [79]. Figure S4 also shows that the maximal absorption of the water peak moves slightly towards a higher number of waves as the Aw increases [79].

To relate the humidity to the area below the water peak, the spectra were superimposed (see Supplementary Figure S4). First, only one peak was used near the 1650 cm^−1^ region to describe the IR water content band. At this initial setting, the center of the water peak was able to move without restriction and each spectrum was treated individually. The energy value has increased from 1643 cm^−1^ for CL-CNC with 0.04% humidity to a value close to 1657 cm^−1^ for biofilm (CS: CL-CNC8:2-20%) with 5.62% humidity, and around 1977 cm^−1^ in the case of biofilm (CS: CL-CNC7:3-50%) with 6.65% humidity.

#### 3.2.4. Optical Properties of the Synthesized Bio-Composites Films 

**Figure 4:**
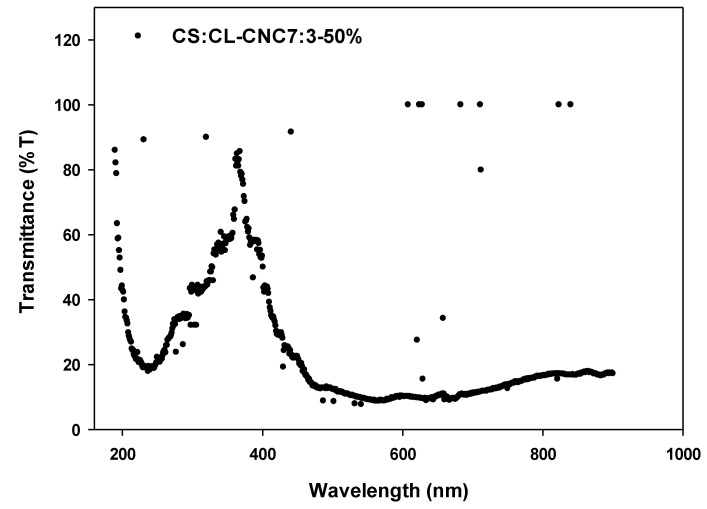
UV-visible transmittance % spectra of CS:CL-CNC 7:3-50% bio-composite film.

The figure transmittance % of CS/CL-CNC7:3-50% bio-composite films on the wavelength range from 190 nm to 900 nm is shown in Figure 4. This figure showed intense transmittance bands in the UV range located at around 212–300 nm but was absent in the visible regions (Figure 4). Thus, these bio-composites could be used in different fields of applications due to being effective UV transmitters, mainly for UV-C radiation from sunlight (100–280 nm), but also for UV-B radiation (280–315 nm). 

### 3.3. Biodegradation of Bio-Composite Films with Enzyme Hydrolysis

Enzymes secreted by microorganisms degrade the thermoplastic starch polymer into its monomeric unit, which serves as a carbon source for microbial growth [80]. The biodegradation of starch was mainly associated with the activity of alpha-amylase and glucoamylase, a particular enzyme for starch hydrolysis that is considered a significant contributor to degradation [81]. Therefore, alpha-amylase is an endo-amylase that hydrolyzes the α-(l,4) starch bonds (amylose and amylopectin) almost at room temperature, whereas glucoamylase is an exo-amylase that hydrolyzes the α-1.4 bonds and the α-(1,6) starch bonds (amylose and amylopectin) near or at room temperature [82]. For this reason, the biodegradability test was carried out using the two types of enzymes (α-amylase and glucoamylase) necessary to break down all the cellulose bonds. 

For testing the condition of activity of the mixture enzyme, the primly test affected the degradation of a known starch solution (10 mg/mL) by the mixture of enzymes (α-amylase and glucoamylase). The objective of this test was to determine the conditions (time, concentration, temperature, etc.) favoring the proper functioning of the enzyme in order to degrade a starch solution of a specific concentration. The attached table (see Supplementary Table S1) summarizes the results. Consequently, the best condition for the release of starch degradation by the combination of two enzymes is an incubation temperature of T = 50 °C, an immersion time of t = 2 h–12 h, and an enzyme volume V = 0.5 mL (31.6 FPU/mL) (see Supplementary Comment).

**Figure 5:**
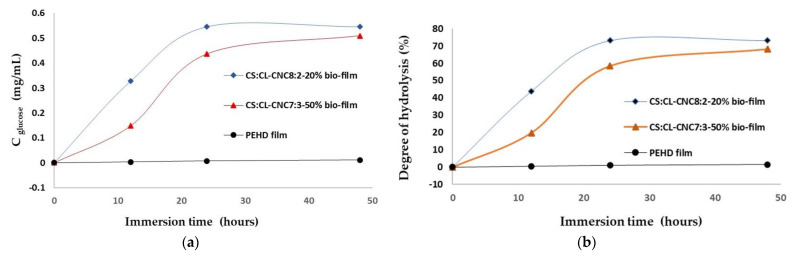
Kinetic bio-degradation of CL-CNC reinforced thermoplastic starch films and HDPE industrial petrochemical films as a reference: (**a**) reduced glucose concentration obtained as a function of the immersion time into the enzyme mixture, and (**b**) the degree of hydrolysis obtained as a function of the time of immersion into the enzymatic mixture.

The enzymatic mixture degraded the prepared CL-CNC-reinforced starch films in a short period of time (24 h) with a hydrolysis degree of 73%, and the degradability of bio-composite film was faster, which is important for the bio-composite film with a ratio of 8:2 (44%) than 7:3 (20%) after 12 h of the enzyme hydrolysis time, as illustrated in the Figure 5a,b. which shows that the enhanced CNC film increases the stiffness of the bio-composite film and delays biodegradation. However, the addition of CNC (30% by weight) increased tensile strength in comparison to CNC-reinforced thermoplastic starch (20% by weight) and reduced biodegradation. For HDPE industrial petrochemical films as a reference, the test of biodegradability was negative and showed no change in the morphology of the film with an enzymatic mixture. 

The results explicate the effect of the ratio of corn thermoplastic starch versus the biodegradation of bio-composite film reinforced by 20% and 30% CNC. However, CNC affects the morphology, flexibility, and kinetic biodegradation of biofilm [83]. The result displayed a decreased degradation rate of CS:CL-CNC 7:3-50% compared to CS:CL-CNC 8:2-20%, since the elevation ratio of starch accelerates the biodegradation rate of the blend compounds. However, when surface-modified starch was replaced by 30% CL-CNC, a significant reduction in the degradation rate was observed from 20% to 40% after 12 h of immersion time in a mixture of enzymes (Figure 5b) [83]. Therefore, CL-CNC-modified thermoplastic starch was an excellent reinforcing agent for thermoplastic starch films, effectively improving the overall performance of the films. This makes CS/CL-CNC bio-composite a potential food packaging material.

## 4. Conclusions

Sustainable bio-composite films based on thermoplastic starch reinforced by CL-CNC with improved properties have been successfully obtained from marine *C. linum* biomass by treatment by alkalinization, bleaching, and acid hydrolysis. The modification of the matrix chemistry (L-CNC/CS) of the bio-composite was analyzed by FTIR and SEM-EDX studies, revealing the formation of hydrogen bonds between CL-CNC and CS, and demonstrating that the best matrix composition is 7:3 (CS:CL-CNC) with 50% glycerol. The optical primary test for this bio-composite film display could be used in different fields of applications due to having an effective UV light transmittance effect, mainly for UV-C radiation from sunlight but also for UV-B radiation. The test of the biodegradability of bio-composite film through an enzymatic mixture (α-amylase and glucoamylase) proves that the CL-CNC-modified thermoplastic corn starch was an excellent reinforcing agent for thermoplastic starch films, effectively improving the overall performance of the films. This makes this bio-composite matrices a potential food packaging material. Due to the promising properties of bio-composites, several new ones are emerging and the assessment of their risks requires an individual approach to each bio-material as a result of concerns regarding the safe use of bio-composites. This study was ameliorated by incorporating a green silver nanoparticle isolated from the same algae biomass in the background to improve the optical and biodegradable properties of biofilms, thereby giving an unambiguous answer for the future application of these biomaterials. 

## Figures and Tables

**Table 1:** Composition of the cellulose nanocrystals, starch, and glycerol used in the investigation of the development of bio-composite.

Sample Code	CS (g)	NaOH (mL)	CL-CNC (g)	CS (g)	Distilled Water (mL)	Glycerol (mL)
CS:CL-CNC 7:3-50%	7.00	40	3.00	7.00	140	90.0
CS:CL-CNC 8:2-20%	8.00	40	2.00	8.00	160	40.0

**Table 3:** Lateral order index (LOI), total crystallinity index (TCI), and hydrogen bonding index (HBI) of the samples CL-MCC and CL-CNC produced from *C. linum*.

	LOI A1430/A908	TCI A1326/A2903	HBI A3350/A1326
CL-MCC	0.994 ± 0.005	1.011 ± 0.006	1.014 ± 0.002
CL-CNC	1.003 ± 0.005	1.023 ± 0.006	1.035 ± 0.002

## Data Availability

The data presented in this study are available on request from the corresponding author.

## Supplementary Materials

The following supporting information can be downloaded at: https://www.mdpi.com/article/10.3390/polym15061542/s1, Figure S1: SEM and EDX analysis showing various steps of cellulose nanocrystals production from *C. linum* with 1000 × magnification and operated at an accelerating voltage of 15 KeV; Figure S2: IR Spectra of the samples (CL-R, CL-A4%, CL-B2.5%, CL-A17.5%, CL-B5%, CL-MCC, and CL-CNC) produced throughout cellulose nanocrystals extract from *C. linum*, (a) (400–4000 cm^−1^), (b) (400–1800 cm^−1^), (c) (2800–3800 cm^−1^), and (d) IR Spectra of the samples CL-MCC and CL-CNC produced from *C. linum*; Figure S3: IR Spectra of the cellulose nanocrystals extract CL-CNC, and of the bio-composite films development (CS: CL-CNC8:2-20 % and CS: CL-CNC7: 3-50 %); Figure S4: Area plot by Sigmaplot (Systat Software Inc., version 12, Bayshore, USA), AW: area of water peak from 1560 to 1800 cm^−1^, AT: total area of IR spectra; Figure S5: The calibration curve for the determination of reductive sugars (glucose) using the DNS method at T = 50°C; Table S1: α-amylase and glucoamylase degradation test of known starch solution (1 mg/mL) at different condition; Table S2: Total area (AT) of IR spectra, and area of water (AW) peak from 1560 to 1800 cm^−1^ determined by Sigmaplot (Systat Software Inc, version 12, Bayshore, USA); Video S1: The real morphology of bio-composites film CS:CL-CNC7:3-50%.
