# Supplementary material for: Part A: Biodegradable Bio-Composite Film Reinforced with Cellulose Nanocrystals from Chaetomorpha linum into Thermoplastic Starch Matrices
Source: Polymers (Basel). 2023 Mar 20;15(6):1542. doi: 10.3390/polym15061542 (PMC10058665; doi:10.3390/polym15061542)
Supplement: Supplementary file 1 [file polymers-15-01542-s001.zip › polymers-2247490-supplementary.pdf]

Supplementary Information

Part A: Biodegradable Bio-Composite Film Reinforced with Cellulose Nanocrystals from *Chaetomorpha linum* into Thermoplastic Starch Matrices

Taghreed Alsufyani [1*†] and Nour Houda M’sakni [1,2*†]

1 Department of Chemistry, College of Science, Taif University, P.O. Box 11099, Taif 21944, Saudi Arabia
2 Laboratory of Interfaces and Advanced Materials (LIMA), Faculty of science, Monastir University, Monastir 5019, Tunisia
* Correspondence: Authors: alsufiyan@tu.edu.sa (T.A.); nour.h@tu.edu.sa (N.H.M)
† These authors contributed equally to this work

Supplementary Figures

SEM	EDX

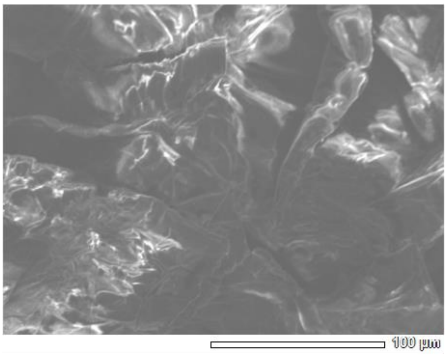

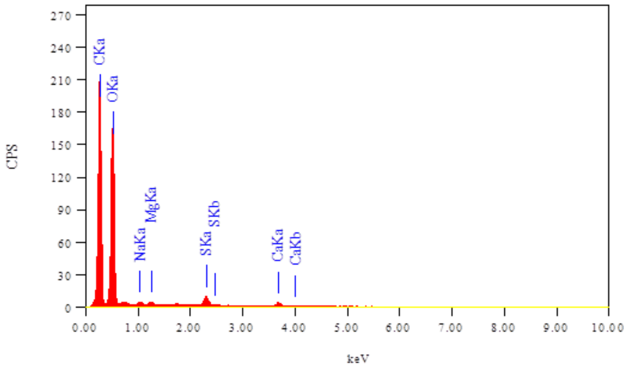

CL-R

ZAF Method Standardless Quantitative Analysis

Fitting Coefficient : 0.1413

Element	(keV)	Mass%	Error%	Atom%	Compound	Mass%	Cation	K
C K	0.277	48.09	0.03	51.61				38.0715
O K	0.525	38.22	0.13	47.65				59.4833
Na K	1.253	3.71	0.09	0.22				0.3809
S K	2.307	2.47	0.09	0.28				0.9997
Cl K	3.690	4.17	0.19	0.23				1.0645
K K	3.690	3.34	0.19	0.23				1.0645
Total		100.00		100.00				

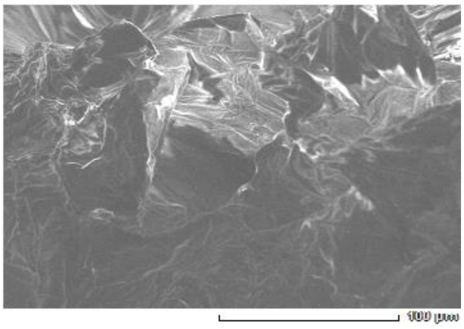

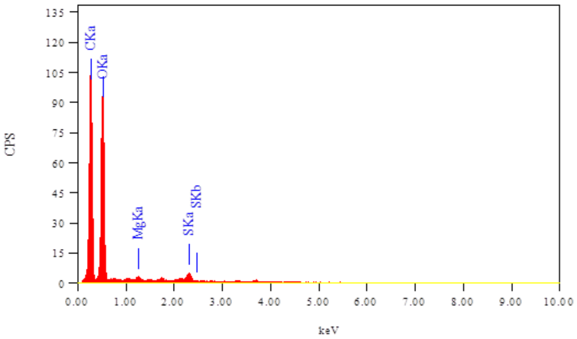

CL-A4%

ZAF Method Standardless Quantitative Analysis

Fitting Coefficient : 0.3887

Element	(keV)	Mass%	Error%	Atom%	Compound	Mass%	Cation	K
C K	0.277	45.09	0.12	52.39				41.6234
O K	0.525	54.13	0.46	47.21				57.2465
Mg K	1.253	0.41	0.23	0.23				0.3971
S K	2.307	0.37	0.15	0.16				0.7331
Total		100.00		100.00				

CL-B2.5%

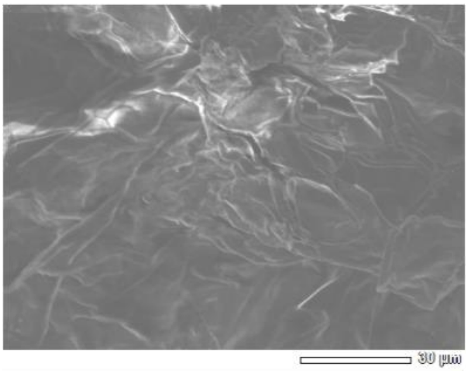

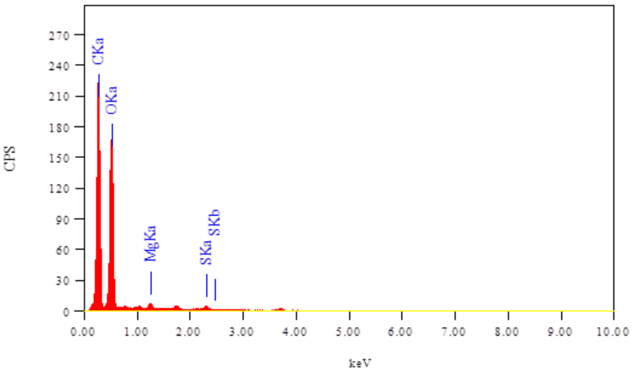

ZAF Method Standardless Quantitative Analysis

Fitting Coefficient : 0.1499

Element	(keV)	Mass%	Error%	Atom%	Compound	Mass%	Cation	K
C K	0.277	47.58	0.03	54.93				43.3673
O K	0.525	51.38	0.14	44.53				55.3139
Mg K	1.253	0.58	0.09	0.33				0.5982
S K	2.307	0.46	0.09	0.20				0.7206
Total			100.00		100.00			

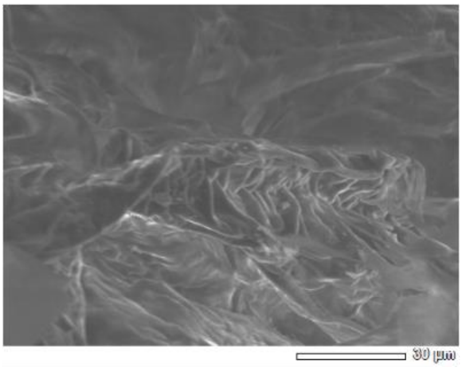

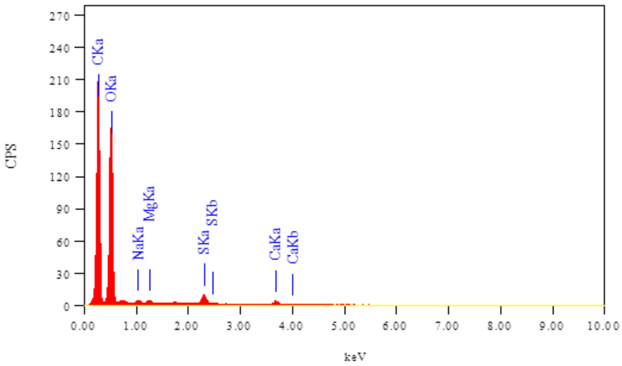

CL-A17.5%

ZAF Method Standardless Quantitative Analysis

Fitting Coefficient : 0.1554

Element	(keV)	Mass%	Error%	Atom%	Compound	Mass%	Cation	K
C K	0.277	46.26	0.03	54.00				40.6083
O K	0.525	51.13	0.14	44.81				55.5560
Na K	1.041	0.42	0.11	0.26				0.4462
Mg K	1.253	0.31	0.09	0.18				0.3258
S K	2.307	1.07	0.09	0.47				1.7294
Ca K	3.690	0.80	0.20	0.28				1.3343
Total			100.00		100.00			

CL-MCC

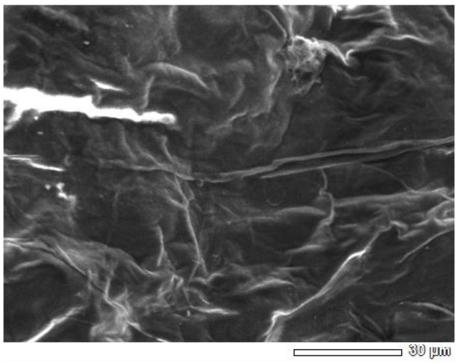

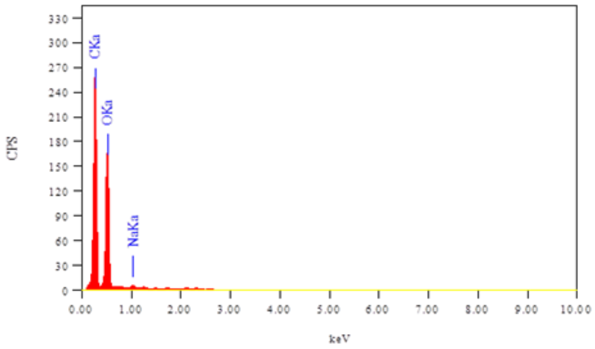

ZAF Method Standardless Quantitative Analysis

Fitting Coefficient : 0.1605

Element	(keV)	Mass%	Error%	Atom%	Compound	Mass%	Cation	K
C K	0.277	49.43	0.03	56.64				47.4487
O K	0.525	50.04	0.16	43.05				52.0107
Na K	1.041	0.52	0.13	0.31				0.5405
Total			100.00		100.00			

CL-CNC

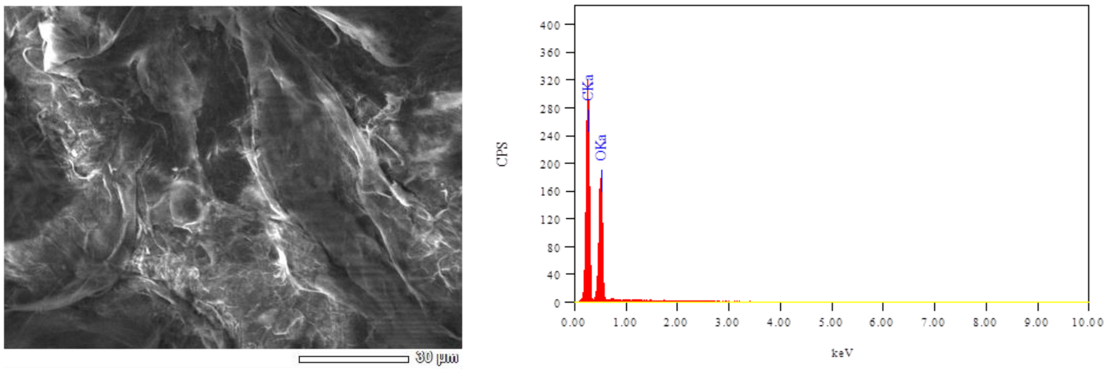

ZAF Method Standardless Quantitative Analysis

Fitting Coefficient : 0.4643

Element	(keV)	Mass%	Error%	Atom%	Compound	Mass%	Cation	K
C K	0.277	51.90	0.13	58.97				51.7236
O K	0.525	48.10	0.70	41.03				48.2764
Total		100.00		100.00				

Figure S1. SEM and EDX analysis showing various steps of cellulose nanocrystals production from *C. linum* with 1000 x magnification and operated at an accelerating voltage of 15 KeV

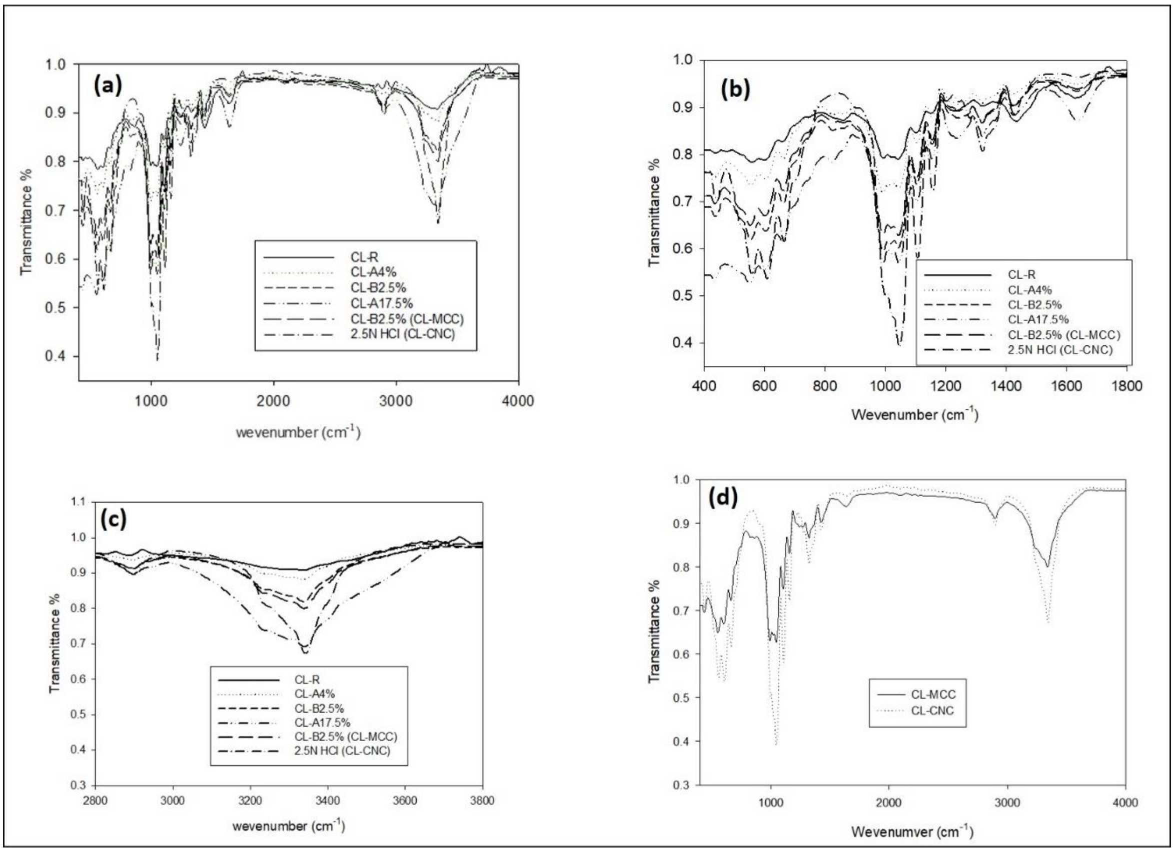

Figure S2. IR Spectra of the samples (CL-R, CL-A4%, CL-B2.5%, CL-A17.5%, CL-B5%, CL-MCC, and CL-CNC) produced throughout cellulose nanocrystals extract from *C. linum*, **(a)** (400-4000 cm^{-1}), **(b)** (400-1800 cm^{-1}), **(c)** (2800-3800 cm^{-1}), and **(d)** IR Spectra of the samples CL-MCC and CL-CNC produced from *C. linum*.

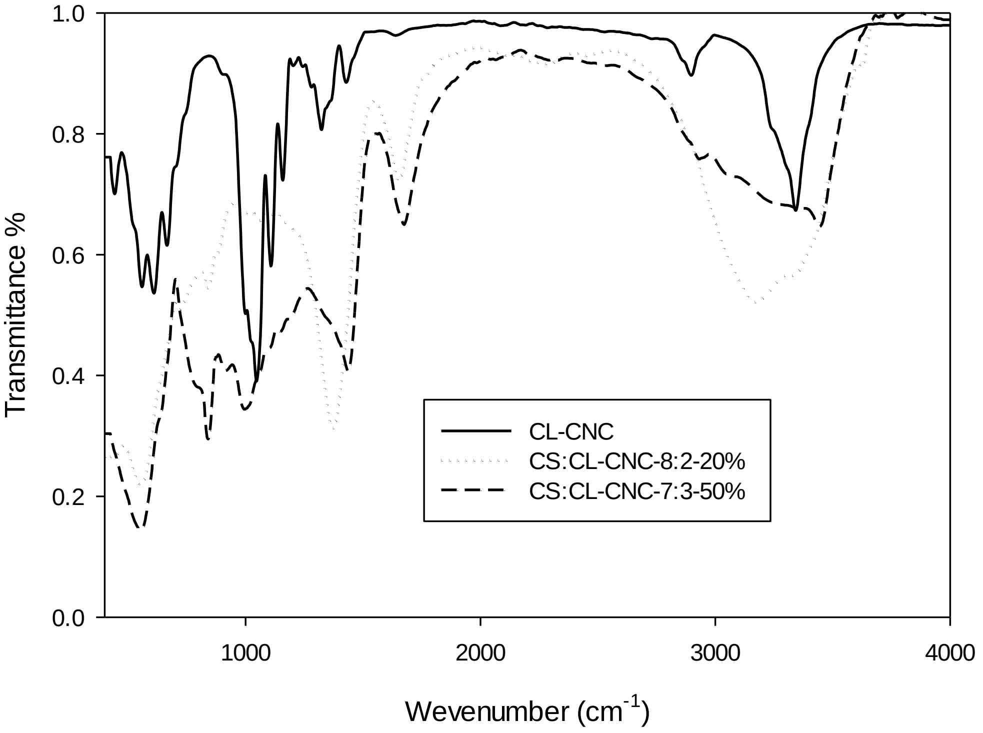

Figure S3. IR Spectra of the cellulose nanocrystals extract CL-CNC, and of the bio-composite films development (CS: CL-CNC8:2-20 % and CS: CL-CNC7: 3-50 %)

Water content calculation

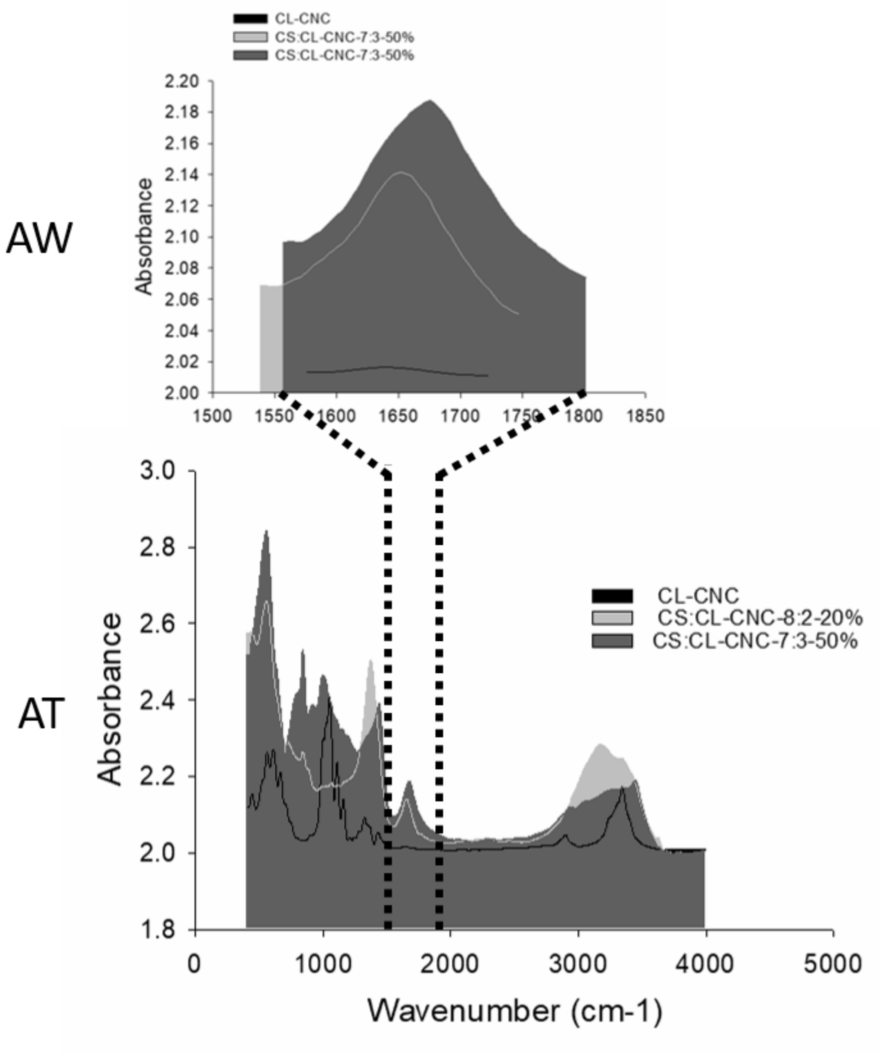

Figure S4. Area plot by Sigmaplot (Systat Software Inc, version 12, Bayshore, USA), AW: area of water peak from 1560 to 1800 cm^{-1}, AT: total area of IR spectra.

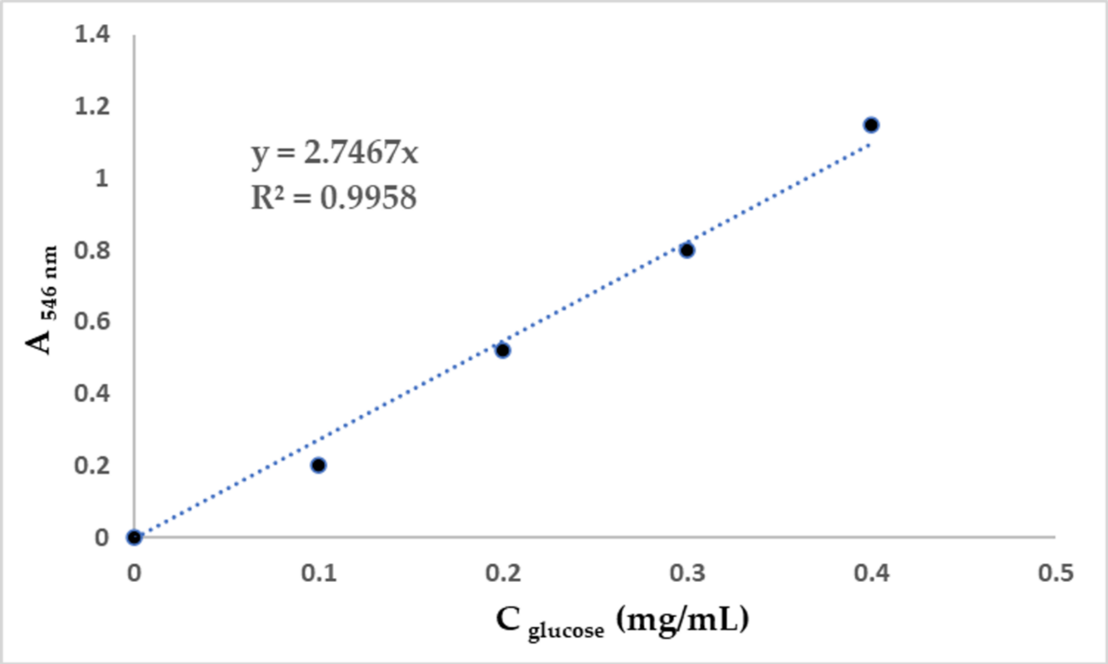

Figure S5. The calibration curve for the determination of reductive sugars (glucose) using the DNS method at T = 50°C.

Supplementary Tables

Table S1. α-amylase and glucoamylase degradation test of known starch solution (1 mg/mL) at different condition

Tubes		1	2	3	4	5	6	7	8
Volume (α-amylase and glucoamylase) (mL)		0.2	0.5	0	0.2	0	0.2	0.5	0
T (°C)		37	37	37	0	0	50	50	50
Fehling liquor	t= 1 h	-	-	-	-	-	-	-	-
	t= 2 h	-	-	-	-	-	-	+	-
	t= 12 h	-	-	-	-	-	+	+	-
Aqueous iodine	t= 1 h	+	+	++	+	+	+	+	+
	t= 2 h	+	+	+	+	+	+	++	+
	t= 12 h	+	+	+	+	+	-	++	-

Note that for tubes 3, 5 and 8, which are subject to (37°C, 0°C and 50°C) and do not have any enzyme, the test is always negative under the action of the Fehling liquor. This proves the absence of glucose in the contents of each tubing. However, we find that with iodine water, the test is positive for both tubes 3 and 5 but negative for tube 8. It can therefore be said that starch underwent a change under the influence of high temperature (50°C), and lost its original structure resulting in the absence of the blue starch indicator color. For tubes 1, 2, and 4 at a temperature T = 37°C (1 and 2), and T = 0°C (tube 4) with an enzyme dose of 0.2 and 0.5, and 0.2 mL respectively, it should be noted at all sampling moment, the starch test is positive and the glucose test is

negative. Starch degradation did not occur. Three hypotheses need to be put forward: the enzyme used is not functional, the temperature is not favorable or we have not yet reached the time required to start the degradation process. Tubes 6 and 7 tested at T = 50°C with an enzymatic volume of 0.2 mL and 0.5 mL, respectively, showed a positive response with Fehling liquor. After 2 h, we start to have a red precipitate for tube 7 and this staining is visible only from 12 h for tube 6. It can be concluded that the first hypothesis is false and that the ideal temperature required for starch degradation is T = 50°C. Consequently, the best condition for the release of starch degradation by the combination of two enzymes is an incubation temperature of T = 50°C, an immersion time of t = 2 h-12 h, and an enzyme volume V = 0.5 mL (31.6 FPU/mL).

Table S2. Total area (AT) of IR spectra, and area of water (AW) peak from 1560 to 1800 cm^{-1} determined by Sigmaplot (Systat Software Inc, version 12, Bayshore, USA).

	**CL-CNC**	**CS:CL-CNC 8:2-20%**	**CS:CL-CNC 7:3-50%**
**AT (UA)**	7385.93	7754.36	7840.99
**AW (UA)**	295.88	436.03	521.16
**Water content (%)**	4.00	5.62	6.65

Supplementary Video

Video S1 video describes the real morphology of bio-composites film CS:CL-CNC7:3-50%
